# Mast Cell Survival and Mediator Secretion in Response to Hypoxia

**DOI:** 10.1371/journal.pone.0012360

**Published:** 2010-08-23

**Authors:** Magdalena Gulliksson, Ricardo F. S. Carvalho, Erik Ullerås, Gunnar Nilsson

**Affiliations:** 1 Clinical Immunology and Allergy Unit, Department of Medicine, Karolinska Institutet, Stockholm, Sweden; 2 Department of Biomedical Sciences and Veterinary Public Health, Swedish University of Agricultural Sciences, Uppsala, Sweden; Fundação Oswaldo Cruz, Brazil

## Abstract

Tissue hypoxia is a consequence of decreased oxygen levels in different inflammatory conditions, many associated with mast cell activation. However, the effect of hypoxia on mast cell functions is not well established. Here, we have investigated the effect of hypoxia *per se* on human mast cell survival, mediator secretion, and reactivity. Human cord blood derived mast cells were subjected to three different culturing conditions: culture and stimulation in normoxia (21% O_2_); culture and stimulation in hypoxia (1% O_2_); or 24 hour culture in hypoxia followed by stimulation in normoxia. Hypoxia, *per se*, did not induce mast cell degranulation, but we observed an increased secretion of IL-6, where autocrine produced IL-6 promoted mast cell survival. Hypoxia did not have any effect on A23187 induced degranulation or secretion of cytokines. In contrast, cytokine secretion after LPS or CD30 treatment was attenuated, but not inhibited, in hypoxia compared to normoxia. Our data suggests that mast cell survival, degranulation and cytokine release are sustained under hypoxia. This may be of importance for host defence where mast cells in a hypoxic tissue can react to intruders, but also in chronic inflammations where mast cell reactivity is not inhibited by the inflammatory associated hypoxia.

## Introduction

Mast cells have an important role in many inflammatory diseases, such as asthma, and they are also involved in response to infections, tumor progression and in conditions related to ischemia [1], [2]. Mast cells are distributed in all vascularised tissues throughout the body and more abundantly in tissues exposed to the environment, i.e., lung, gut and skin. This makes them one of the first cells exposed to allergens, pollutants and pathogens [3].

Oxygen concentrations may vary in cells and tissue but when the gradient of partial pressure (*p*O_2_) drops below the normal level it is denoted as hypoxic [4]. As a result of the reduced oxygen levels in tissue the metabolism is shifted to consume less oxygen and at the same time erythropoiesis and angiogenesis are induced to restore the limited blood supply [5]. Hypoxia is a prominent feature of inflamed tissues; including tumors, myocardial infarcts, atherosclerotic plaques, lung of asthmatics, healing wounds and sites of bacterial infections. Several of these conditions are also associated with increased number of mast cells [6]. In contrast to the effect of hypoxia on macrophage functions that is well documented [7], the effect of hypoxia on mast cell functions is poorly investigated.

In this study we have investigated the effect of hypoxia (1% O_2_) *per se* on human mast cell survival, degranulation and cytokine secretion. In addition, we have analysed the effect of hypoxia on mast cell reactivity using external factors known to activate mast cells under certain conditions, i.e., mast cell activation in chronic inflammation and tumours (CD30 activation) [8], bacteria membrane component stimulation (LPS) and increase in calcium (calcium ionophore A23187). One important question to clarify is if hypoxia is triggering mast cells and if mast cells become unresponsive to other triggers during hypoxic conditions. Retained mast cell responsiveness under hypoxia would be of importance for their protective role in health and disease.

## Results

### Mast cell survival is sustained under hypoxia

**Figure 1:**
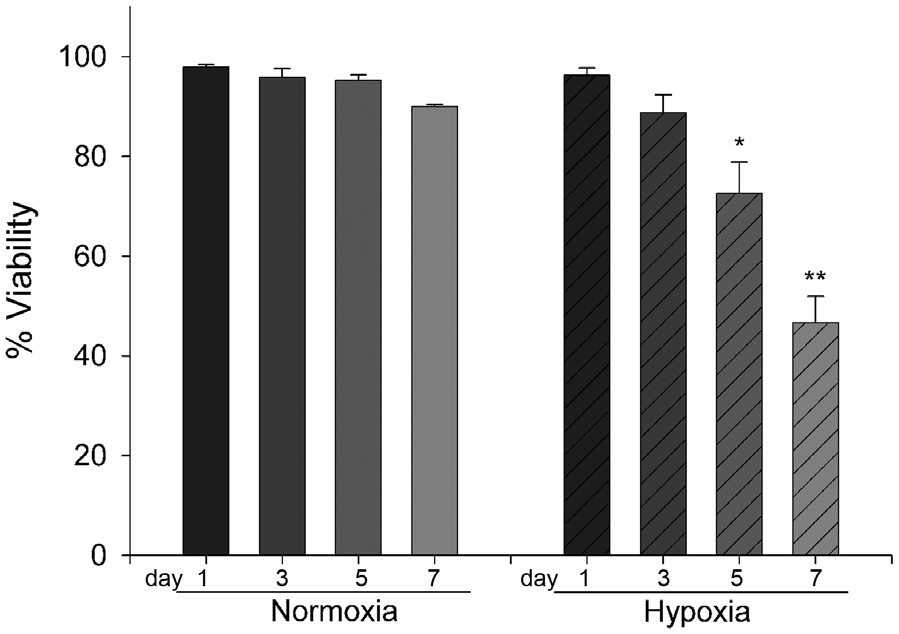
Mast cell viability. Survival of CBMC cultured for 1 to 7 days in) normoxia (21% O_2_) or hypoxia (1% O_2_). Cell viability was calculated using trypan blue exclusion. Two different donors, n = 4, mean ± SEM. * p<0-05 and ** p<0.01 compared to day 1.

First we investigated the effect of hypoxia (1% O_2_) on mast cell viability. We found that cells cultured in hypoxia sustain a high viability for up to three days. After five days in hypoxia, a significant drop to 73% viability was observed (*P* = 0.024), which was further decreased to 47% at day seven (Fig. 1). These results suggest that CBMC are viable in hypoxia for several days and consequently data from cells cultured up to five days in hypoxia should not be biased by cell apoptosis or necrosis.

### The effect of hypoxia on mast cell degranulation and cytokine secretion

**Figure 2:**
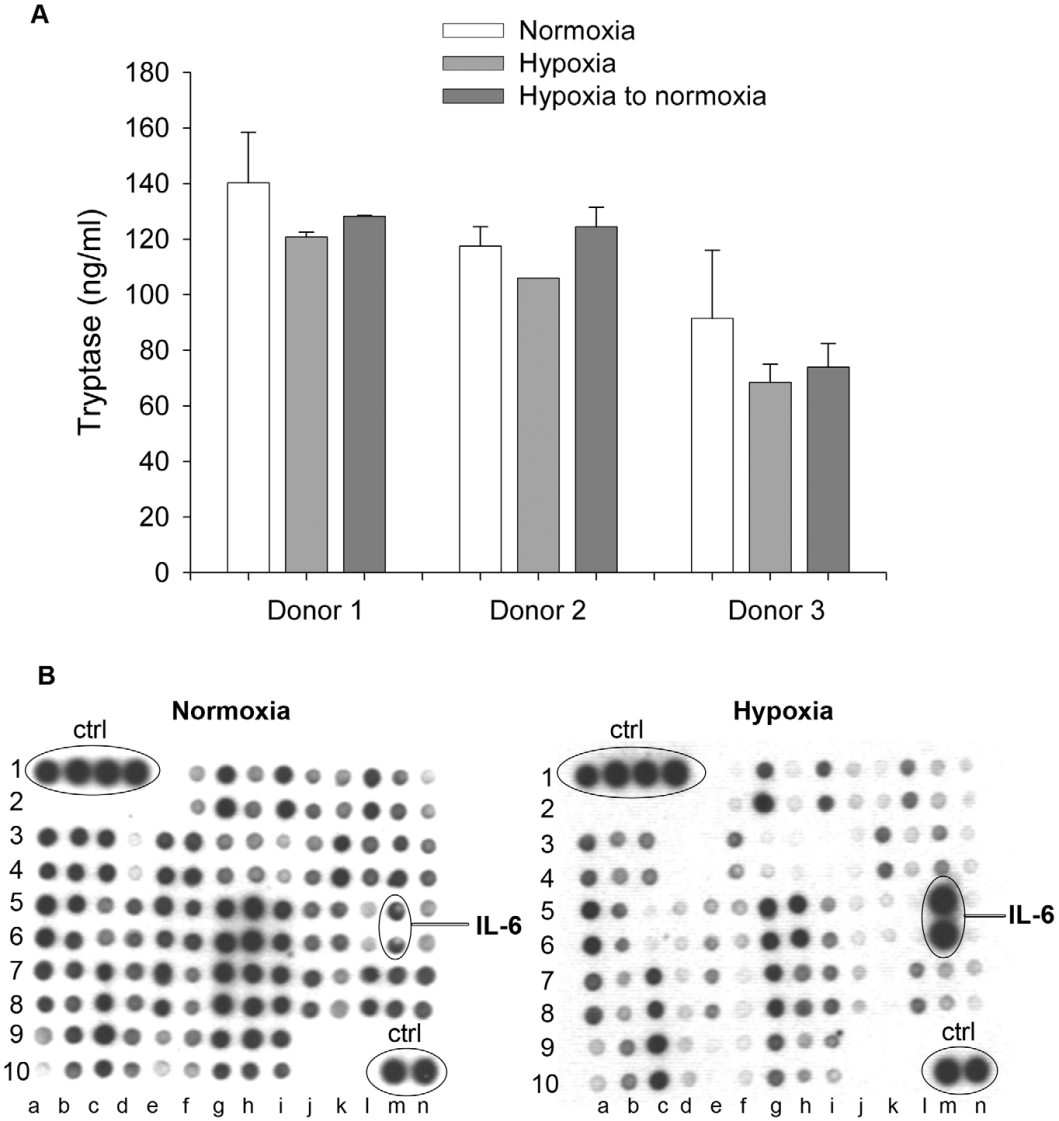
Mast cell mediator release. **A** Mast cell tryptase release before or after hypoxia, three different donors, n = 3, mean ± SEM. **B** Antibody array: Protein secretion in response to normoxia and hypoxia. The identity of some of the proteins included in the array is indicated. A complete description of the array is found in Table 1.

We next studied if hypoxia *per se* induces mast cell degranulation and release of granule mediators such as tryptase. As shown in figure 2A we could not observe any increase in the release of tryptase in cells cultured in hypoxia for 24h compared to normoxia. We also pre-incubated the cells in hypoxia for 24h and then transferred them to normoxia to investigate how reoxygenation for 24h affected the cells. We could not observe any difference in release of tryptase if cells were pre-treated in hypoxia compared to normoxia. Thus, hypoxia does not induce mast cell degranulation by itself (Fig. 2A).

Hypoxia activates HIF-1α which regulates the transcription of several cytokines and growth factors in, e.g., macrophages [7]. We therefore measured the effect of hypoxia on cytokine secretion from mast cells deprived of IL-6 for two days and cultured in hypoxic conditions for 24 h. The deprivation was performed to avoid contamination of exogenous added IL-6 to the culture medium. An antibody array was used to screen for candidate cytokines that could be regulated by hypoxia. As shown in figure 2B the spontaneous secretion of several proteins was reduced by hypoxia, whereas only IL-6 appeared to be induced. The identity of the spots in the array is provided in Table 1. Our results suggest that hypoxia *per se* induces secretion of a limited number of cytokines where the secretion of IL-6 was the most pronounced of those analyzed.

**Table 1:** Identity of the spots in the antibody array used in figure 2.

	a	b	c	d	e	f	g	h	i	j	k	l	m	n
**1**	+ctrl	+ctrl	+ctrl	+ctrl	blank	Ang	BDNF	BLC	BMP-4	BMP-6	CK β 8-1	CNTF	EGF	Eotaxin
**2**	−ctrl	−ctrl	−ctrl	−ctrl	blank	Ang	BDNF	BLC	BMP-4	BMP-6	CK β 8-1	CNTF	EGF	Eotaxin
**3**	Eotaxin-2	Eotaxin-3	FGF-6	FGF-7	Flt-3 Lig	Fractalkine	GCP-2	GDNF	GM-CSF	I-309	IFN-γ	IGFBP-1	IGFBP-2	IGFBP-4
**4**	Eotaxin-2	Eotaxin-3	FGF-6	FGF-7	Flt-3 Lig	Fractalkine	GCP-2	GDNF	GM-CSF	I-309	IFN-γ	IGFBP-1	IGFBP-2	IGFBP-4
**5**	IGF-1	IL-10	IL-13	IL-15	IL-16	IL-1α	IL-1β	IL-1ra	IL-2	IL-3	IL-4	IL-5	IL-6	IL-7
**6**	IGF-1	IL-10	IL-13	IL-15	IL-16	IL-1α	IL-1β	IL-1ra	IL-2	IL-3	IL-4	IL-5	IL-6	IL-7
**7**	Leptin	LIGHT	MCP-1	MCP-2	MCP-3	MCP-4	M-CSF	MDC	MIG	MIP-1δ	MIP-3α	NAP-2	NT-3	PARC
**8**	Leptin	LIGHT	MCP-1	MCP-2	MCP-3	MCP-4	M-CSF	MDC	MIG	MIP-1δ	MIP-3α	NAP-2	NT-3	PARC
**9**	PDGF-BB	RANTES	SCF	SDF-1	TARC	TGF-β1	TGF-β3	TNF-α	TNF-β	blank	blank	blank	blank	blank
**10**	PDGF-BB	RANTES	SCF	SDF-1	TARC	TGF-β1	TGF-β3	TNF-α	TNF-β	blank	blank	blank	+ctrl	+ctrl

### HIF-1α is activated under hypoxic conditions

**Figure 3:**
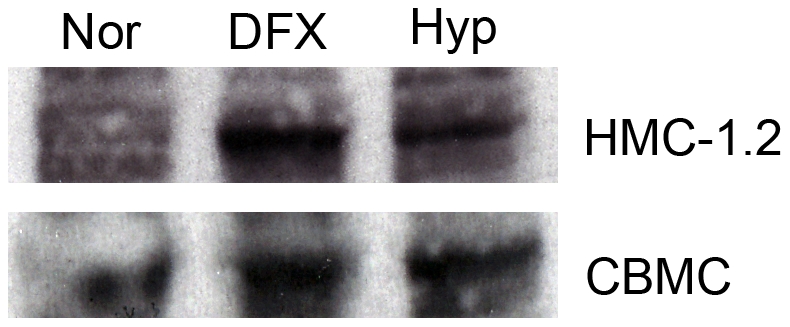
HIF-1α accumulation under hypoxia. HMC-1.2 and CBMC were cultured for 24 h under hypoxia or normoxia. HIF-1α accumulation was determined by western blot. DFX was used as a positive control. The figure is representative of three independent experiments.

Under hypoxic conditions several cellular mechanisms can be activated. The transcription factor HIF-1α is stabilised under low oxygen concentrations and can thus activate a variety of genes involved in the control of cellular metabolism. A mast cell derived cell line, HMC-1.2, and CBMC were cultured for 24h, both under normoxic and hypoxic conditions. As a positive control, we used deferoxamide (DFX), which is a well-described stabiliser of HIF-1α. Both hypoxic conditions and DFX induced an increased accumulation of the HIF-1α protein in both mast cell types tested (Fig. 3).

### Autocrine IL-6 promotes mast cell survival in hypoxia

**Figure 4:**
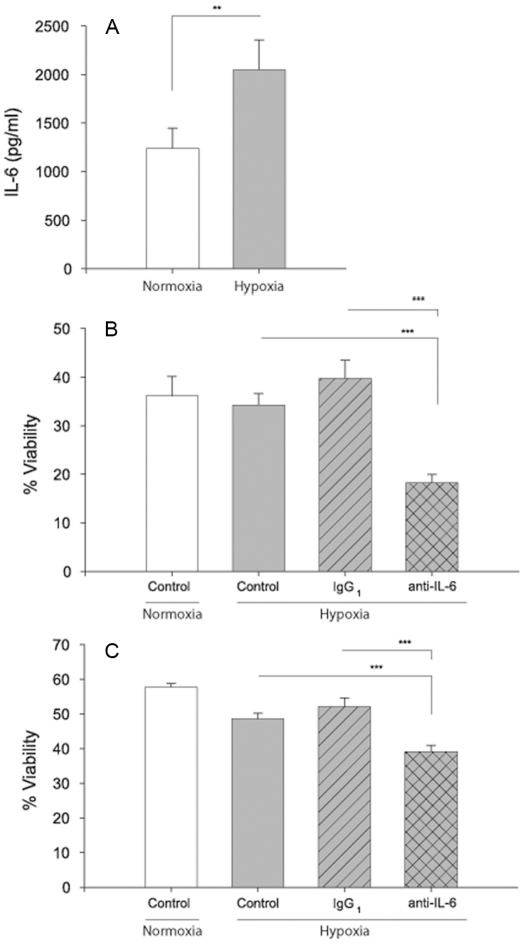
IL-6 is a mast cell survival factor. **A** Mast cell IL-6 secretion after 96 h culture in hypoxia. **B–C** Mast cell viability after 96 h culture in hypoxia as analysed with B trypan blue exclusion (B) and Annexin V, PI staining (C). Cells were treated with a neutralising anti-IL-6 or isotype control antibody (1.0 ug/ml). n = 3, mean ± SEM.** *P*<0.01, *** *P*<0.001.

We first confirmed that IL-6 secretion is induced by hypoxia. As shown in figure 4A, increased levels of IL-6 could be measured in supernatants from mast cells cultured in hypoxia for 96h, as compared to normoxia. In addition, other cytokines were analyzed using a CBA flex kit. The levels of FGF_2_, MIP-1β, IL-1β, angiogenin and GM-CSF were below the detection limit (data not shown). VEGF and TNF secretion was not consistent in the different donors analysed (data not shown). Since IL-6 is a survival factor for human mast cells [9]–[11] we next investigated if IL-6 released from hypoxic mast cells could promote mast cell survival in an autocrine fashion. Mast cells were deprived of SCF and IL-6 for two days before they were cultured for 96h in hypoxia and normoxia in the presence of an IL-6 neutralisation antibody or isotype control. IL-6 neutralisation induced apoptosis with significantly decreased cell viability in hypoxia compared to cultures treated with the isotype control antibody, as assessed by trypan blue exclusion (Fig. 4B) and PI/Annexin V staining (Fig. 4C). Under normal oxygen conditions, the neutralizing antibody did not have any effect on cell survival compared to isotype control (data not shown).

### Mast cells retain reactivity to different stimuli during and after hypoxia

**Figure 5:**
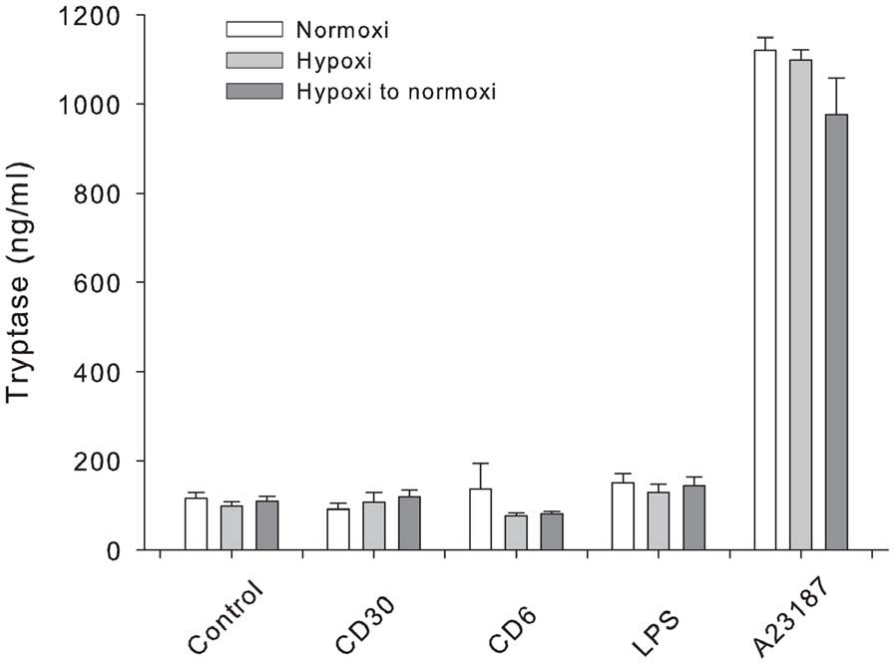
Mast cell degranulation. Tryptase release 24 h after stimulation in response to different stimuli. Cells were cultured in 24 well plates and treated with stimuli in normoxia, hypoxia or after hypoxia for 24 h. Supernatants were analysed for the content of tryptase in three different donors, n = 3, mean ± SEM.

One of the most important features of mast cells is their capacity to react to different stimuli. Since mast cells are distributed in tissues that may reach transient hypoxia, we next investigated if mast cells retained reactivity after hypoxia treatment *in vitro*. To examine if mast cell reactivity is influenced by hypoxia, cells were subjected to stimuli known to act on different signalling pathways under three different conditions: 1) stimulation 24 h in normoxia; 2) stimulation 24 h in hypoxia; and 3) incubation 24 h in hypoxia and then transferred to normoxia and stimulated for 24 hr. The cells were stimulated with A23187, LPS or CD30, and the release of tryptase and IL-8 were measured as markers for reactivity towards stimuli. As shown in figure 5, only A23187 significantly induced degranulation assessed as release of tryptase compared to control. There was no difference in A23187 reactivity between cells stimulated before normoxia, hypoxia or those cultured in hypoxia for 24 before stimulation in normoxia (Fig. 5).

**Figure 6:**
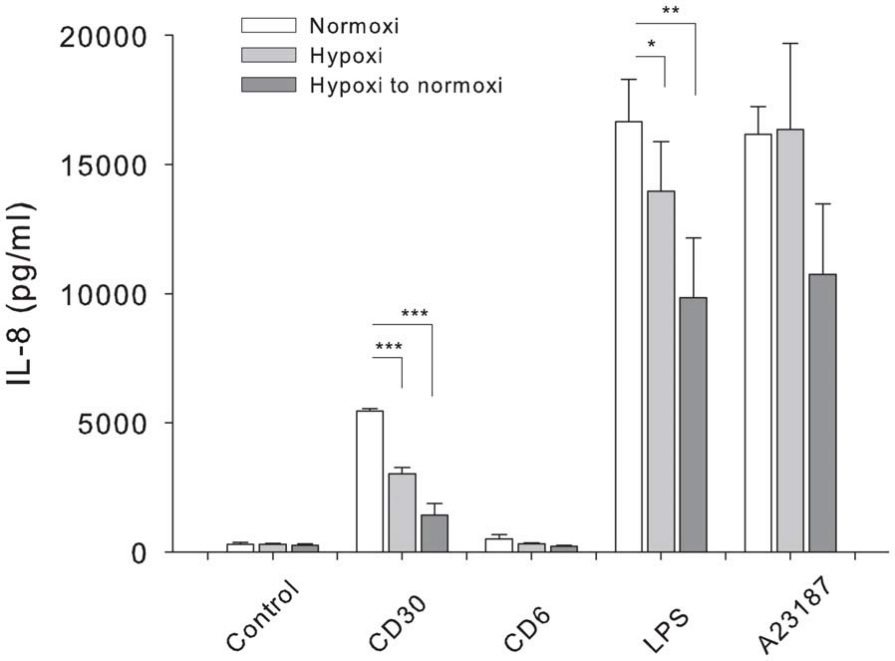
IL-8 secretion. IL-8 release 24 h after stimulation in response to different stimuli. Cells were cultured in 24 well plates and treated with stimuli in normoxia, hypoxia or after hypoxia for 24 h and then transferred to normoxia and stimulated for 24 h. Supernatants were analysed for the content of IL-8 in three different donors, n = 3, mean ± SEM and ^*^ *P*<0.05, **P<0.01, ***P<0.001.

Mast cells express CD30 ligand/CD153 and we have previously shown that activation by CD30-Fc fusion protein induces a degranulation-independent release of IL-8 [8]. As a negative control for this experiment we used a CD6-Fc fusion protein. We also measured the release of IL-8 after treatment with LPS and A23197. All three stimuli induced IL-8 secretion both in normoxia, hypoxia and in reoxygenated cultures (Fig. 6). Upon CD30 and LPS treatment the release was significantly attenuated in the hypoxic cultures and the reoxygenated cultures, compared to normoxia (Fig. 6). Thus, although mast cells seem to be less responsive to activating stimuli after hypoxia and even less responsive after reoxygenation, as observed from the IL-8 release data, they are still reactive.

## Discussion

Mast cells play important roles in several physiological and pathological processes [1]. Several chronic inflammations are associated with an accumulation and activation of mast cells, such as in asthma, rheumatic diseases and tumours [12]–[14]. During these conditions, the tissues are also typically exposed to prolonged or intermittent hypoxia. Although this implies that mast cells are viable and reactive under hypoxic conditions, very little data exists today that supports this hypothesis [15]–[17].

Our data suggests that mast cells are unresponsive to transient hypoxia. First, CBMC were viable for several days in hypoxia (Fig. 1), secondly there was no spontaneous degranulation in response to hypoxia (Fig. 2A), and thirdly mast cells were still reactive to stimuli under hypoxia. This confirms that mast cells are stable to certain physical environmental changes [18], [19]. In order to exclude that the results were biased by pH differences we also analysed the pH of the medium in hypoxia and normoxia after 24–96 h and found no differences in pH levels between the two conditions (data not shown).

In other cell types, persistent hypoxia can result in cell death [20] or mediate a shift in mediator release and disturb cell functions [21], [22]. In macrophages, hypoxia regulates cell functions in inflammation and in contrast to our results macrophages respond rapidly to hypoxia by altering gene expression and release of cytokines [7].

Our data shows that hypoxia provokes IL-6 secretion in mast cells (Fig. 2B). IL-6 is important for mast cell proliferation [23] and to promote human mast cell survival [9], [10]. In a previous study, neutralisation of IL-6 in human lung mast cell cultures significantly decreased the cell viability after seven days in normoxia [11], implicating that mast cells may induce their own survival in an autocrine/paracrine fashion. Further, stimulation with IgE promoted the survival of human lung mast cells and this relation was dose dependent and hindered by an anti-IL-6 antibody [11]. In the present study we found that anti-IL-6 treatment of mast cells in hypoxia decreased their survival. Thus, our data support previous reports that IL-6 produced by mast cells can act as an autocrine/paracrine survival factor.

One may expect that hypoxia *per se* would induce secretion of a subset of cytokines and growth factors, e.g., those regulated by HIF-1α. In our study we found an accumulation of HIF-1α when cells were cultured under hypoxia (fig. 3), but we did not see a consistent increase in cytokine secretion (fig. 2B). Recently, interesting data supporting the notion that mast cells are rather unresponsive to hypoxia was reported, where it was described that additional signalling pathways are needed to induce HIF-1α expression in human mast cells [24]. Ionomycin, C5a and substance P, but not mastoparan, was shown to induce HIF-1α expression. Furthermore, the induction was dependent on a calcineurin-NFAT binding site in the HIF1A promoter region for accumulation of HIF-1α and induction of target genes [24]. This may explain our low response to hypoxia compared to normoxia and also the low response in combination with other stimuli.

The only cytokine found to be significantly induced by hypoxia was IL-6. IL-6 is not a classical hypoxia-induced gene, but is probably regulated by other transcription factors under hypoxic conditions. Hypoxia-mediatied induction of IL-6 in myocytes has been shown to be regulated by NFκB and NF-IL6 [25]. Furthermore, IL-6 was induced in human mast cell line HMC-1 upon treatment with DFX, an induction that was partly inhibited by pre-treatment with a NFκB-inhibitor [16]. There might also be other pathways involved in the regulation of cellular responses to hypoxia. One example is change in redox balance that might effect the activity of the cells. Another interesting finding is that mast cell degranulation appears not be affected by redox changes induced by hypoxia [26]. However, the effect on cytokine synthesis and secretion has not been studied.

Since mast cells are stable in hypoxia we believe that they may have an important function in innate responses to inflammation and bacteria, virus, and parasite infections [27]. Though mast cells often respond to different stimulus with release of mediators, they also tend to be very stable to environmental factors that affect many other haematopoietic cells. For example, both human and mouse mast cells are unaffected by gamma radiation and can be activated after radioactive exposure with an equal amount of release compared to non radiated cells [18]. Thus, mast cells constitute an important defence mechanism to pathogens even after radiation. Furthermore, ultraviolet B irradiation had no effect on release of granulae stored mediators of human mast cells [19]. In line with these data we hypothesise that mast cells are refractory to effects of transient hypoxia with no or very little impact on mediator secretion.

Hypoxia is related to conditions with limited blood flow as in inflammatory diseases such as asthma and joint inflammation but also in tumour progression [4]. Subjects with airway mucous plugging can reach low oxygen levels downstream in the bronchial tree and hypoxic conditions in the lung may result in hypertrophy and increased airway smooth muscle accumulation [21]. For patients with an active lung disease mast cells may be important for the clearance of pathogens. The reactivity of mast cells under low oxygen pressure would then be essential for host defence under these conditions.

There are also several studies on the role of mast cells in ischemia and reperfusion injury, e.g., myocardial infarction [28]. Mast cell degranulation has been detected in models of ischemia-reperfusion models, but this mast cell activation might be a result of complement activation of mast cells [29]. Thus, in these models mast cells might not be activated directly by hypoxia or the reperfusion, but through indirect mechanisms.

As outlined in previous studies, mast cells can be very selective in their mediator secretion [30] and our results show that this is also true for stimuli acting on different signalling pathways under hypoxia. A23187, but neither CD30 nor LPS activation, induced release of tryptase (Fig. 4). The effect of A23187 was not affected by hypoxia. Cytokine production was in some cases influenced by hypoxia in combination with different stimuli. In response to CD30 and LPS stimulation, the release of IL-8 was decreased in hypoxia and after reoxygenation compared to normoxia (Fig. 5).

CD30 is expressed in Hodgkin lymphoma, atopic dermatitis and psoriasis, diseases where mast cells are the predominant cell type expressing CD153 [8], [31]. The effect of hypoxia on CD30-mediated stimulation was more pronounced compared to the effect on LPS or A23187 reactivity. This implies that CD30-activation of mast cells in hypoxic tissues is attenuated in these diseases.

Our results show that mast cells survive and can be activated during hypoxia. Thus, mast cells retain reactivity to external triggering factors. This suggests that mast cells can play important roles in host defence even in a tissue with low oxygen pressure. Understanding the effect of hypoxia on mast cell functions is critical to understand the role of mast cells in diseases, such as cancers and asthma that are affected by hypoxia.

## Methods

### Preparation of cord blood derived mast cells (CBMC)

Isolation and preparation of cells was performed as previously described [32]. Briefly, mononuclear cells were isolated from heparinised human umbilical cord blood. CD34^+^ cells were selected from the mononuclear cells with MACS MicroBeads (Miltenyi Biotech, Germany) and cultured for 3–5 weeks in StemPro serum free medium (Invitrogen life technologies, USA) (week 1, 20 000 cells/ml, following weeks, 2000 00 cells/ml) supplemented with 10 ng/ml IL-3 (on the first week of culture) (Peprotech, UK), human recombinant stem cell factor (SCF), 100 ng/ml and 10 ng/ml human recombinant IL-6 (both kindly provided by Amgen, Thousand Oaks, USA). When cells were >95% tryptase positive they were transferred to RPMI 1640 medium (Sigma, USA) with supplements including heat inactivated FCS (10%), SCF (100 ng/ml) and IL-6 (10 ng/ml) and kept at a 1×10^6^ cells/ml cell density. Medium was changed weekly and these cells were designated as cord blood derived mast cells (CBMC). Cultures were kept in 37°C, 5% CO_2_ and 1% (hypoxia) or 21% (normoxia) O_2_ (Sanyo incubator). For all treatments, cells were cultured in 24 well plates before collection of supernatants and cell analysis. If nothing else stated, cells were stimulated in RPMI medium with supplements including 10% FCS.

### Culture of HMC-1.2 cells

The human mast cell line HMC-1.2 cells was maintained as previously described [33]. Briefly, cells were kept in IMDM, L-glutamine (HyClone, South Logan, UT, USA) medium supplemented with 10% heat-inactivated FCS (Life Technologies, Paisley, UK), α-monothioglycerol, 100 IU/ml penicillin and 50 µg/ml streptomycin (Sigma).

### Cell death detection

Cell viability and apoptosis were analysed by trypan blue exclusion and/or by fluorescence-activated cell sorting (FACS) using propidium iodide (PI) and FITC-conjugated Annexin V (Becton Dickinson, Sweden) staining of cells. Data was analysed by FACScalibur and CellQuest software and viable cells were calculated as percent of total cell amount.

### Western blot

CBMC and HMC-1.2 were cultured for 24h under normoxia or hypoxia at 1×10^6^ cells/ml cell density, total cells 4×10^6^. As a positive control, cells were treated with 100 µM DFX (Sigma). Cells were lysed using 100 µl of a solution of SDS+DTT. Samples were loaded on a NuPage 4–12% Bis-Tris gel (Invitrogen) and run for 1h at 200 V. Gel was blotted to a Hybond-ECL nitrocellulose membrane (Amersham, Sweden). Membrane was blocked with 5% fat-free milk in TBS-T 0.1% for 1h at room temperature, followed by overnight incubation with anti-human HIF-1α-HRP (Novus Bio, UK) 1∶500 in TBS-T-5% BSA solution. Protein was detected using Lumiglo (Cell Signalling Technology) and luminescence detected using Hyperfilm-ECL (Amersham).

### Analysis of degranulation

To assess the degree of degranulation, the release of tryptase was measured by ImmunoCAP™ (Phadia AB, Uppsala, Sweden).

### Antibody array

Secretion of multiple cytokines in cell supernatants was detected by human cytokine antibody Arrays VI and VII (Chemicon, Sweden). Briefly, cell supernatants were added to a solid array membrane coated with antibodies against specific cytokines. All cell incubations were performed with 0.2% BSA instead of serum for minimisation of false positive data. A biotin conjugated primary antibody together with HRP-Streptavidin substrate was used for detection of analytes. The membranes were exposed to x-ray film and detected by a chemiluminescence imaging system. A complete description of the cytokines analyzed with the antibody array can be found in Table 1.

### Analysis of cytokines and chemokines

Cell supernatants were analysed with ELISA for the content of IL-8 (BioSource, Camarillo, USA) and IL-6 (R&D systems, Stockholm, Sweden). The assays were performed with a double sandwich ELISA as described by the manufacturer. A CBA flex set assay of seven different beads detecting cytokines and chemokines (FGF_2_, MIP-1β, IL-1β, VEGF, TNF, angiogenin and GM-CSF) was used (Becton Dickinson, Sweden). Preparation of beads was performed according to manufacturer's instructions. Data was collected with FACSAria and analysis was performed with FACSDiva and FCAPArray software (Beckton Dickinson).

### Neutralisation of IL-6

Cells were cultured in RPMI with supplements but without IL-6, or without IL-6 and SCF, for two days before further treatment. After two days, soybean protease inhibitor (100 µg/ml, Sigma-Aldrich, Sweden), anti-human IL-6 (1.0 µg/ml, R&D systems, Sweden) or IgG_1_ isotype control (1.0 µg/ml) were added. Cells were incubated for 24, 48 and 96 h in hypoxia or normoxia.

### Treatment with CD30, CD6, LPS and A23187

Cells were treated with immobilized CD30 (10 µg/ml), or CD6 (10 µg/ml, R&D systems), LPS (1 µg/ml, Sigma-Aldrich) or A23187 (0.5 µM, Sigma-Aldrich) for 24 h. All reagents were dissolved in PBS or dH_2_O with the exception of A23187 which was used with a maximum of 0.5% DMSO. Cells were cultured in supplemented RPMI with 0.2% BSA.

### Statistical analyses

SigmaStat software was used to perform the statistical analysis. Comparisons between more than two groups were made with parametric tests (One Way Repeated Analysis of Variance). Further pair wise comparisons were performed with a student's paired t-test. Difference was regarded as significant if *P*<0.05. All data are expressed as mean ± SEM.
